# The prognostic nutritional index improves risk stratification for acute pulmonary embolism

**DOI:** 10.1016/j.isci.2025.114623

**Published:** 2026-01-02

**Authors:** Shuangping Li, Shenshen Huang, Wei Wang, Jing Zhang, Bo Chen, Kelei Guo, Chenglong Ma, Shuaihui Hou, Pengfei Gao, Yimin Mao

**Affiliations:** 1 College of Clinical Medicine, Department of Respiratory and Critical Care Medicine, The First Affiliated Hospital, Henan University of Science and Technology, Luoyang, Henan, China; 2 Department of Respiratory and Critical Care Medicine, The First Affiliated Hospital, Henan University of Science and Technology, Luoyang, Henan, China; 3 Medical Records and Statistics Office, The First Affiliated Hospital, Henan University of Science and Technology, Luoyang, Henan, China; 4 Operating Room, The First Affiliated Hospital, Henan University of Science and Technology, Luoyang, Henan, China; 5 Department of Respiratory and Critical Care Medicine, Yanshi People’s Hospital, Luoyang, Henan, China

**Keywords:** Health sciences

## Abstract

Risk stratification guides management in acute pulmonary embolism (APE), yet current models have limitations. We investigated the Prognostic Nutritional Index (PNI) as a potential biomarker to refine risk assessment. Analyzing 1,163 discovery, 208 internal-validation, and 212 external-validation APE patients, we found that a higher PNI was independently associated with lower 30-day and in-hospital mortality after multivariable adjustment. Incorporating PNI into the European Society of Cardiology (ESC) risk model improved its predictive performance for 30-day mortality. Crucially, a PNI ≤42.5 effectively stratified intermediate-risk patients, identifying subgroups with 4.7- and 6-fold higher 30-day mortality in the intermediate-low- and intermediate-high-risk categories, respectively. These findings position PNI as a simple, valuable tool for enhancing precision in APE risk stratification.

## Highlights

• Low PNI predicts substantially higher mortality risk in APE
• Adding PNI to the 2019 ESC model improves its 30-day mortality prediction accuracy
• PNI stratifies intermediate-risk APE patients for intensified care

## Graphical abstract

**Figure:**
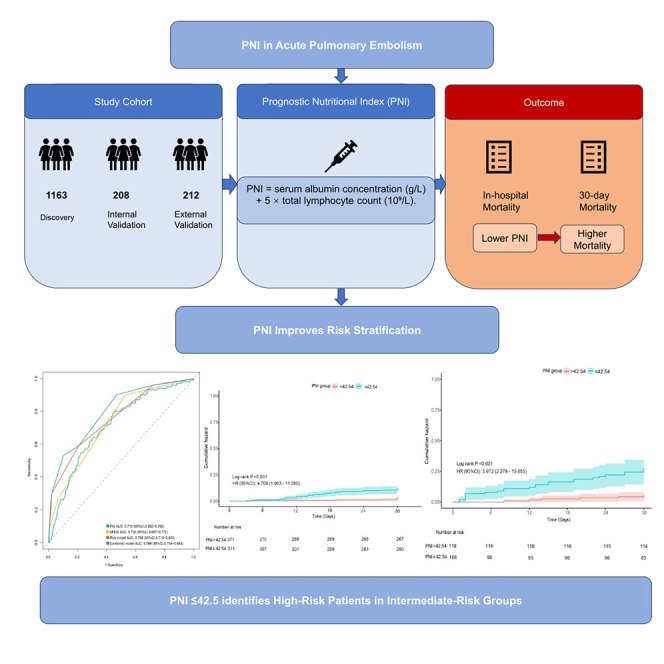

## Introduction

Pulmonary embolism (PE) leads to high mortality among hospitalized patients and represents one of the greatest threats to global health care.^1^^,^^2^ Driven by rapid population aging and the growing burden of comorbidities, including cardiovascular disease and cancer, hospital admissions for PE have been steadily increasing. With growing awareness of acute pulmonary embolism (APE) and advances in diagnostic and therapeutic strategies, the 30-day mortality rate of APE has shown a declining trend; however, the proportion of related deaths remains high. In China, the mortality rate decreased from 25.1% in 1997 to 8.6% in 2008,^3^ whereas in the United States, the 30-day mortality rate declined from 12.7% in 1999 to 9.4% in 2015.^4^ Appropriate diagnosis and management based on optimal assessment and risk stratification can reduce the mortality of APE. The Pulmonary Embolism Severity Index (PESI)^5^ and its simplified version (sPESI)^6^ have been widely applied for risk stratification in hospitalized patients with APE and have been incorporated into the European Society of Cardiology (ESC) guidelines.^7^ However, the strength of PESI and sPESI lies primarily in identifying low- to intermediate-risk patients with APE, whereas their ability to discriminate intermediate- to high-risk patients remains limited.^8^ The ESC 2019 guidelines thus proposed a multi-parameter prognostic model that classifies patients into four risk categories based on hemodynamic status: sPESI score, right ventricular dysfunction (RVD), and cardiac biomarkers.^7^

The prognostic value of nutritional status, which reflects overall patient health, has been demonstrated in a variety of diseases, including acute coronary syndrome,^9^ stable coronary artery disease,^10^ heart failure,^11^ and acute ischemic stroke.^12^^,^^13^ However, a simple, inexpensive, and reliable indicator of nutritional status is still required for APE to predict short-term prognosis. The Prognostic Nutritional Index (PNI), originally developed by Onodera et al.,^14^ is an immunonutritional score calculated from the peripheral lymphocyte count and serum albumin concentration. The PNI provides a comprehensive assessment of both immune and nutritional status^15^^,^^16^ and has been shown to have prognostic value in patients with decompensated liver cirrhosis,^17^ chronic kidney disease,^18^ type 2 diabetes,^19^ and cardiovascular diseases.^20^^,^^21^

Only one previous study has shown that a lower PNI on admission is a predictor of higher in-hospital mortality in patients with APE.^22^ However, the association between the PNI and 30-day mortality in patients with APE has not yet been investigated. Therefore, we aimed to evaluate the short-term prognostic value of the PNI and incorporate it into existing risk stratification models, with the goal of improving the accuracy of 30-day mortality prediction. This may provide a refined tool for risk stratification in patients with APE, serve as a cornerstone of comprehensive APE management, and ultimately improve patient outcomes and standards of care.

## Results

### Study population and basic clinical characteristics

**Figure 1:**
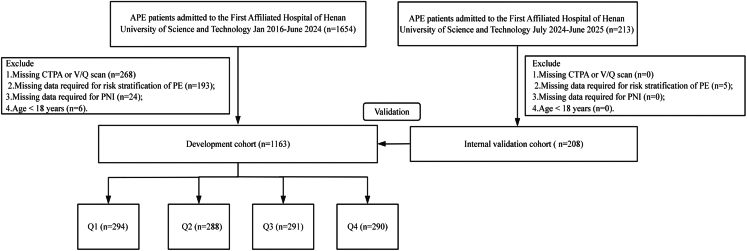
Flowchart of patient selection

Based on the inclusion and exclusion criteria, a total of 1,163 patients were ultimately enrolled in the discovery cohort, and 208 patients were included in the internal validation cohort (Figure 1). A total of 212 participants were included in the external validation cohort, as detailed in Figure S1. In the discovery cohort, patients were subsequently categorized into four quartiles according to their PNI values: Q1 (PNI ≤37.90, *n* = 294), Q2 (37.90 < PNI ≤42.95, *n* = 288), Q3 (42.95 < PNI ≤47.90, *n* = 291), and Q4 (PNI >47.90, *n* = 290). The baseline characteristics of the discovery cohort are summarized in Table 1. Overall, 618 patients (53.14%) were male, and 545 (46.86%) were female, with a median age of 70 years. According to the ESC risk stratification for APE, 302 patients (25.97%) were classified as low-risk, 582 (50.04%) as intermediate-low-risk, 225 (19.35%) as intermediate-high-risk, and 54 (4.64%) as high-risk. Within 30 days, 100 patients (8.60%) died, and the in-hospital mortality was 106 (9.11%). In the internal validation cohort, 102 patients (49.04%) were male and 106 (50.96%) were female, with a median age of 71 years. Within 30 days, 15 patients (7.21%) died. According to the ESC risk stratification, 56 patients (26.92%) were classified as low-risk, 116 (55.77%) as intermediate-low-risk, 25 (12.02%) as intermediate-high-risk, and 11 (5.29%) as high-risk, as shown in Table S2. In the external validation cohort, 51.89% (110/212) of patients were male, with a median age of 63 years. The 30-day mortality was 11.79% (25/212); 15.57% of patients were low-risk, 46.70% intermediate-low-risk, 33.02% intermediate-high-risk, and 4.72% high-risk (Table S3).

**Table 1:** Basic characteristics of the study participants stratified by the PNI

Variables	Total (*n* = 1163)	Q1 (*n* = 294)	Q2 (*n* = 288)	Q3 (*n* = 291)	Q4 (*n* = 290)	*p*
**Demographic variables**						

Age, years	70.00 (59.00, 77.00)	72.00 (64.00, 80.00)	71.00 (61.00, 78.00)	70.00 (61.00, 77.00)	63.00 (53.25, 73.00)	<0.001
Weight, kg	84.00 (69.00, 89.00)	86.00 (73.25, 89.00)	80.00 (65.00, 87.00)	79.00 (68.00, 89.00)	86.00 (70.00, 89.00)	0.414
Male, n (%)	618 (53.14)	183 (62.24)	163 (56.60)	132 (45.36)	140 (48.28)	<0.001

**Vital signs**						

Heart rate, bpm	86.00 (78.00, 100.00)	91.50 (80.00, 105.75)	88.50 (78.00, 100.00)	84.00 (76.00, 96.00)	82.00 (73.25, 96.00)	<0.001
SBP, mmHg	124.00 (110.50, 137.00)	121.00 (109.00, 136.00)	123.50 (111.00, 136.00)	124.00 (112.00, 137.00)	125.00 (112.00, 137.00)	0.408
RR, bpm	20.00 (19.00, 22.00)	20.00 (19.00, 23.00)	20.00 (19.00, 22.00)	20.00 (19.00, 21.00)	20.00 (18.00, 21.00)	<0.001
PO_2_, mmHg	71.60 (61.35, 88.80)	71.30 (61.20, 88.00)	73.00 (60.83, 90.78)	70.60 (59.75, 85.95)	72.00 (63.73, 89.75)	0.329
PCO_2_, mmHg	35.60 (31.40, 39.90)	35.30 (31.70, 39.80)	36.20 (31.20, 40.00)	34.50 (30.90, 38.80)	36.20 (32.00, 40.38)	0.030

**Comorbidities**						

Sepsis, n (%)	63 (5.42)	29 (9.86)	19 (6.60)	8 (2.75)	7 (2.41)	<0.001
Cancer, n (%)	208 (17.88)	53 (18.03)	62 (21.53)	47 (16.15)	46 (15.86)	0.260
Hypertension, n (%)	434 (37.32)	88 (29.93)	117 (40.62)	122 (41.92)	107 (36.90)	0.012
CAD, n (%)	208 (17.88)	46 (15.65)	54 (18.75)	56 (19.24)	52 (17.93)	0.678
Heart failure, n (%)	28 (2.41)	9 (3.06)	11 (3.82)	2 (0.69)	6 (2.07)	0.079
CKD, n (%)	40 (3.44)	18 (6.12)	9 (3.12)	4 (1.37)	9 (3.10)	0.016
Diabetes, n (%)	158 (13.59)	39 (13.27)	33 (11.46)	50 (17.18)	36 (12.41)	0.197
CPD, n (%)	71 (6.10)	22 (7.48)	27 (9.38)	13 (4.47)	9 (3.10)	0.007

**Laboratory tests**						

WBC count, ×10^9^/L	7.57 (5.83, 10.23)	8.17 (6.09, 11.65)	7.55 (5.49, 9.84)	7.21 (5.57, 9.65)	7.52 (6.26, 10.17)	0.006
HGB level, g/L	118.00 (96.00, 135.00)	106.70 (85.00, 125.00)	113.00 (90.00, 130.25)	121.00 (101.00, 136.00)	129.00 (111.70, 141.00)	<0.001
Platelet count, ×10^9^/L	208.00 (160.00, 270.00)	199.00 (140.25, 265.75)	201.50 (149.00, 274.50)	212.00 (166.00, 258.50)	220.00 (183.00, 276.50)	0.004
Neutrophil count, ×10^9^/L	5.48 (3.81, 7.96)	6.57 (4.66, 10.17)	5.82 (3.76, 7.98)	5.02 (3.44, 7.47)	4.96 (3.65, 7.06)	<0.001
Lymphocyte count, ×10^9^/L	1.28 (0.86, 1.77)	0.80 (0.53, 1.15)	1.08 (0.81, 1.43)	1.41 (1.10, 1.77)	1.88 (1.51, 2.33)	<0.001
Albumin level, g/L	36.70 (32.40, 40.10)	29.80 (27.30, 31.60)	35.25 (33.30, 37.10)	38.30 (36.70, 39.90)	42.10 (40.10, 43.90)	<0.001
Lactate level, mmol/L	1.40 (1.00, 2.00)	1.40 (1.00, 2.00)	1.40 (0.96, 1.96)	1.40 (1.00, 1.90)	1.40 (1.00, 2.00)	0.628
D-dimer level, mg/L	3.43 (1.70, 8.04)	4.20 (1.99, 10.22)	4.11 (1.79, 9.15)	3.23 (1.74, 6.50)	2.66 (1.06, 5.75)	<0.001
Total bilirubin level, μmol/L	12.20 (8.80, 17.28)	11.40 (8.40, 17.78)	12.30 (8.97, 17.22)	12.70 (9.15, 17.50)	12.15 (9.00, 16.60)	0.489
Direct bilirubin level, μmol/L	3.80 (2.60, 5.60)	4.30 (3.00, 7.18)	4.00 (2.70, 5.60)	3.60 (2.35, 5.35)	3.40 (2.30, 4.88)	<0.001
ALT level, U/L	23.00 (14.00, 41.00)	25.50 (15.00, 53.75)	21.50 (14.00, 37.00)	22.00 (14.00, 37.50)	24.00 (15.00, 38.90)	0.065
AST level, U/L	21.00 (16.00, 30.00)	25.00 (17.00, 40.00)	21.00 (15.00, 29.25)	20.00 (15.00, 28.50)	21.00 (16.00, 27.00)	<0.001
Glucose level, mmol/L	5.80 (4.88, 7.38)	6.26 (4.90, 8.30)	5.70 (4.82, 7.27)	5.79 (4.85, 7.29)	5.70 (4.92, 6.92)	0.176
BUN level, mmol/L	5.30 (3.90, 7.00)	6.12 (4.40, 8.65)	5.20 (3.89, 6.90)	4.90 (3.60, 6.45)	5.20 (3.92, 6.50)	<0.001
Creatinine level, μmol/L	67.00 (55.16, 82.00)	64.91 (53.00, 84.00)	67.50 (56.75, 83.00)	65.25 (54.32, 81.00)	67.50 (57.00, 79.00)	0.459

**Severity**						

sPESI>0, n (%)	651 (55.98)	205 (69.73)	176 (61.11)	155 (53.26)	115 (39.66)	<0.001
Positive cardiac troponin, n (%)	338 (29.06)	104 (35.37)	86 (29.86)	74 (25.43)	74 (25.52)	0.024
Positive NT-proBNP, n (%)	573 (49.27)	182 (61.90)	145 (50.35)	134 (46.05)	112 (38.62)	<0.001
RVD, n (%)	573 (49.27)	156 (53.06)	142 (49.31)	150 (51.55)	125 (43.10)	0.082
ESC risk stratification, n (%)						<0.001
Low risk	302 (25.97)	41 (13.95)	64 (22.22)	82 (28.18)	115 (39.66)	
Intermediate-low risk	582 (50.04)	172 (58.50)	154 (53.47)	140 (48.11)	116 (40.00)	
Intermediate-high risk	225 (19.35)	51 (17.35)	62 (21.53)	61 (20.96)	51 (17.59)	
High risk	54 (4.64)	30 (10.20)	8 (2.78)	8 (2.75)	8 (2.76)	

**Therapy**						

rt-PA, n (%)	30 (2.58)	14 (4.76)	5 (1.74)	5 (1.72)	6 (2.07)	0.056
Catheter-based therapy, n (%)	29 (2.49)	10 (3.40)	3 (1.04)	10 (3.44)	6 (2.07)	0.189

**Outcome**						

In-hospital mortality, n (%)	106 (9.11)	61 (20.75)	25 (8.68)	13 (4.47)	7 (2.41)	<0.001
30-day mortality, n (%)	100 (8.60)	56 (19.05)	24 (8.33)	13 (4.47)	7 (2.41)	<0.001

SBP, systolic blood pressure; RR, respiratory rate; PCO_2_, partial pressure of carbon dioxide; PO_2_, partial pressure of oxygen; WBC, white blood cell; HGB, hemoglobin; BUN, blood urea nitrogen; ALT, alanine aminotransferase; AST, aspartate aminotransferase; sPESI, simplified pulmonary embolism severity index; CAD, coronary artery disease; CPD, chronic pulmonary disease; CKD, chronic kidney disease; NT-proBNP, N-terminal pro–B-type natriuretic peptide; RVD, right ventricular dysfunction; ESC, European Society of Cardiology; rt-PA, recombinant tissue-type plasminogen activator.

Table 1 presents the baseline characteristics of the study population stratified by PNI quartiles. Patients in the lowest PNI group (Q1) were generally older, were more likely to be males, and had higher heart rates and respiratory rates and lower systolic blood pressure than those in the higher PNI groups (*p* < 0.05). Several comorbidities were more prevalent in Q1, including sepsis, hypertension, chronic kidney disease (CKD), and chronic pulmonary disease (CPD) (*p* < 0.05). Laboratory findings revealed that Q1 patients had lower hemoglobin and albumin levels; lower platelet and lymphocyte counts; higher white blood cell and neutrophil counts; and higher D-dimer, direct bilirubin, AST, and BUN levels (*p* < 0.05). Regarding disease severity, patients in Q1 exhibited a higher prevalence of an sPESI >0, as well as positive cardiac troponin and elevated NT-proBNP levels compared to the higher PNI groups (*p* < 0.05). According to the ESC risk stratification, low-risk patients were predominantly concentrated in Q4, while high-risk patients were mainly distributed in Q1 (*p* < 0.05). In terms of outcomes, both in-hospital mortality and 30-day mortality were significantly higher in Q1 than in Q4 (20.75% vs. 2.41% and 19.05% vs. 2.41%, respectively) (*p* < 0.05).

### Kaplan-Meier survival curve analysis

**Figure 2:**
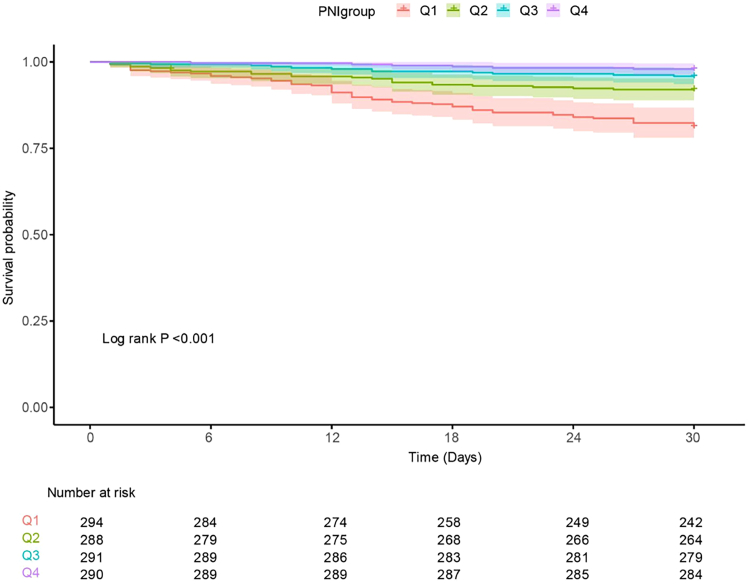
Kaplan-Meier survival curves for 30-day survival in different PNI groups of patients Kaplan-Meier analysis of overall survival stratified by PNI quartiles (Q1–Q4). Survival probability differed significantly across the four groups over time (log rank test, *p* < 0.001). The corresponding table displays the number of patients at risk at predefined time points for each quartile.

Kaplan-Meier survival analysis demonstrated significant differences in survival probabilities among the four PNI quartile groups (log rank *p* < 0.001; Figure 2). Patients in the lowest PNI quartile (Q1) exhibited the poorest survival, with a markedly steeper decline in survival probability than those in the higher quartiles. In contrast, patients in the highest PNI quartile (Q4) had the most favorable outcomes, maintaining the highest survival probability throughout the follow-up period.

### Relationships between the PNI and 30-day and in-hospital all-cause mortality

**Figure 3:**
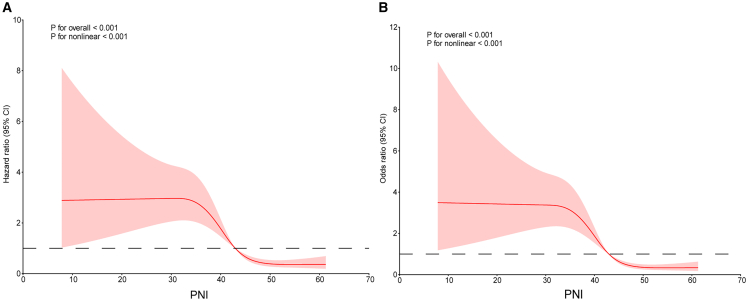
Dose-response relationships of the PNI with clinical outcomes using RCS (A) 30-day mortality. (B) In-hospital mortality. The solid curves represent the estimated associations, with the dashed line indicating the reference level (HR or OR = 1). Both models showed significant overall associations and nonlinearity (*p* < 0.001 for both).

We used an restricted cubic spline (RCS) regression model to evaluate the risk of mortality, revealing a nonlinear relationship between the PNI and both 30-day mortality and in-hospital mortality, with the inflection point in both models occurring at 42.95 (*P* for overall <0.001; *P* for nonlinearity <0.001). Below this threshold, lower PNI values were associated with a markedly elevated risk, whereas higher PNI values conferred a protective effect (Figure 3). In model 3, no multicollinearity was detected among the variables (all the variance inflation factors [VIFs] were <5). In the analysis of 30-day mortality, the fully adjusted model (model 3) revealed that each one-unit increase in the PNI was associated with a 6% reduction in the risk of death (hazard ratio [HR] 0.94, 95% confidence interval [CI] 0.91–0.97; *p* < 0.001). When analyzed by quartiles, when Q1 was used as a reference, the HRs for Q2–Q4 were 0.42, 0.22, and 0.12 (all *p* < 0.001) in the unadjusted model; after full adjustment (model 3), the associations remained significant, with HRs of 0.57 (95% CI 0.34–0.96, *p* = 0.034), 0.44 (95% CI 0.22–0.87, *p* = 0.019), and 0.21 (95% CI 0.08–0.55, *p* < 0.001), respectively. In the analysis of in-hospital mortality, the fully adjusted model (model 3) demonstrated that each one-unit increase in the PNI was associated with a 6% reduction in the risk of death (OR 0.94, 95% CI 0.91–0.97; *p* < 0.001). Quartile analysis demonstrated that, compared with those for Q1, the ORs for Q2–Q4 were 0.36, 0.18, and 0.09 (all *p* < 0.001) in the unadjusted model; after full adjustment, the protective association persisted, with ORs of 0.45 (95% CI 0.25–0.83, *p* = 0.010), 0.31 (95% CI 0.14–0.70, *p* < 0.001), and 0.13 (95% CI 0.04–0.39, *p* < 0.001), respectively, as shown in Table 2. These findings indicate that higher PNI levels are independently associated with lower 30-day and in-hospital mortality, even after adjustment for demographic, clinical, and laboratory covariates.

**Table 2:** Association of the PNI with 30-day and in-hospital mortality

Variable	Model 1		Model 2		Model 3	
	HR (95% CI)	*p*	HR (95% CI)	*p*	HR (95% CI)	*p*
**30-day mortality**						

Continuous	0.95 (0.93–0.96)	<0.001	0.95 (0.93–0.96)	<0.001	0.94 (0.91–0.97)	<0.001
Q1	Ref.		Ref.		Ref.	
Q2	0.42 (0.26–0.68)	<0.001	0.42 (0.26–0.68)	<0.001	0.57 (0.34–0.96)	0.034
Q3	0.22 (0.12–0.40)	<0.001	0.23 (0.12–0.42)	<0.001	0.44 (0.22–0.87)	0.019
Q4	0.12 (0.05–0.25)	<0.001	0.13 (0.06–0.29)	<0.001	0.21 (0.08–0.55)	<0.001

**In-hospital mortality**						

Continuous	0.94 (0.92–0.95)	<0.001	0.94 (0.92–0.96)	<0.001	0.94 (0.91–0.97)	<0.001
Q1	Ref.		Ref.		Ref.	
Q2	0.36 (0.22–0.60)	<0.001	0.37 (0.22–0.61)	<0.001	0.45 (0.25–0.83)	0.010
Q3	0.18 (0.10–0.33)	<0.001	0.19 (0.10–0.37)	<0.001	0.31 (0.14–0.70)	<0.001
Q4	0.09 (0.04–0.21)	<0.001	0.11 (0.05–0.25)	<0.001	0.13 (0.04–0.39)	<0.001

Model 1 was an unadjusted model.

Model 2 adjusted for age, sex, and weight.

Model 3 adjusted for model 2 and heart rate, RR, SBP, PO_2_, PCO_2_, HGB, platelet count, neutrophil count, D-dimer, ALT, AST, lactate, creatinine, Bun, sPESI, positive cardiac troponin, positive NT-proBNP, RVD, sepsis, heart failure, CPD, hypertension, CAD, diabetes, CKD, and cancer.

### Subgroup analysis

In subgroup analyses, higher PNI was consistently associated with lower 30-day and in-hospital mortality across most patient subgroups, regardless of sex, age, hypertension status, coronary artery disease status, or type 2 diabetes status. However, the strength of this association varied by clinical context. Specifically, no significant associations were detected in patients with low- or high-risk APE, sepsis, cancer, heart failure, CKD, or CPD (all *p* > 0.05; Table 3). Stratified analyses by age, sex, sepsis status, cancer status, hypertension status, heart failure status, CKD status, and CPD status revealed no significant interactions (all *p* values for interactions >0.05), indicating overall consistency across these subgroups. Notably, stronger protective effects of a higher PNI were observed in patients with CAD (30-day HR 0.88, 95% CI 0.84–0.93; in-hospital odds ratio [OR] 0.87, 95% CI 0.80–0.94) than in those without CAD (30-day HR 0.95, 95% CI 0.94–0.97; in-hospital OR 0.94, 95% CI 0.92–0.96). Similarly, compared with patients without diabetes, patients with type 2 diabetes had greater protective effects (30-day HR 0.86, 95% CI 0.80–0.92; in-hospital OR 0.85, 95% CI 0.78–0.93) (30-day HR 0.95, 95% CI 0.94–0.97; in-hospital OR 0.94, 95% CI 0.92–0.96). Significant interactions were detected in both groups, suggesting that the PNI may confer enhanced protective effects in individuals with concomitant CAD and type 2 diabetes. With respect to ESC risk stratification, the PNI was significantly associated with mortality in the intermediate-risk group, but the association was weaker and nonsignificant in low- and high-risk patients. These findings suggest that while the PNI generally predicts mortality risk in patients with APE, its prognostic value may be modified by specific comorbidities and clinical risk categories.

**Table 3:** Subgroup analyses for the association between the PNI and 30-day and in-hospital mortality

Variables	HR (95% CI)	*p*	*P* for interaction	OR (95% CI)	*p*	*P* for interaction
All patients	0.95 (0.93–0.96)	<0.001		0.94 (0.92–0.95)	<0.001	
ESC risk stratification			0.127			0.247
Low risk	0.98 (0.87–1.11)	0.802		0.98 (0.88–1.09)	0.709	
Intermediate-low risk	0.95 (0.93–0.98)	<0.001		0.95 (0.92–0.98)	<0.001	
Intermediate-high risk	0.91 (0.86–0.96)	<0.001		0.89 (0.83–0.95)	<0.001	
High risk	0.98 (0.94–1.02)	0.337		0.95 (0.89–1.01)	0.109	
Age			0.355			0.087
<65 years	0.96 (0.93–0.98)	<0.001		0.95 (0.92–0.98)	<0.001	
≥65 years	0.94 (0.92–0.96)	<0.001		0.92 (0.89–0.95)	<0.001	
Sex			0.611			0.286
Male	0.94 (0.92–0.97)	<0.001		0.93 (0.90–0.95)	<0.001	
Female	0.95 (0.93–0.98)	<0.001		0.95 (0.92–0.98)	<0.001	
Sepsis			0.680			0.343
No	0.95 (0.93–0.97)	<0.001		0.94 (0.92–0.96)	<0.001	
Yes	0.93 (0.87–1.00)	0.055		0.90 (0.82–0.99)	0.023	
Cancer			0.289			0.213
No	0.94 (0.93–0.96)	<0.001		0.93 (0.91–0.95)	<0.001	
Yes	0.96 (0.93–1.00)	0.069		0.96 (0.92–1.00)	0.050	
Hypertension			0.462			0.332
No	0.95 (0.93–0.97)	<0.001		0.94 (0.92–0.96)	<0.001	
Yes	0.94 (0.91–0.97)	<0.001		0.92 (0.88–0.96)	<0.001	
Coronary artery disease			0.015			0.026
No	0.95 (0.94–0.97)	<0.001		0.94 (0.92–0.96)	<0.001	
Yes	0.88 (0.84–0.93)	<0.001		0.87 (0.80–0.94)	<0.001	
Heart failure			0.298			0.238
No	0.95 (0.93–0.96)	<0.001		0.93 (0.91–0.95)	<0.001	
Yes	0.99 (0.90–1.10)	0.906		1.00 (0.89–1.11)	0.934	
Chronic kidney disease			0.805			0.832
No	0.95 (0.93–0.97)	<0.001		0.94 (0.92–0.96)	<0.001	
Yes	0.94 (0.87–1.02)	0.130		0.94 (0.87–1.03)	0.180	
Chronic pulmonary disease			0.756			0.776
No	0.95 (0.93–0.96)	<0.001		0.94 (0.92–0.96)	<0.001	
Yes	0.93 (0.83–1.05)	0.235		0.92 (0.80–1.05)	0.202	
Diabetes			0.009			0.015
No	0.95 (0.94–0.97)	<0.001		0.94 (0.92–0.96)	<0.001	
Yes	0.86 (0.80–0.92)	<0.001		0.85 (0.78–0.93)	<0.001	

ESC, European Society of Cardiology.

### Predictive performance of the PNI, sPESI, ESC risk stratification, and combined model for 30-day mortality

**Figure 4:**
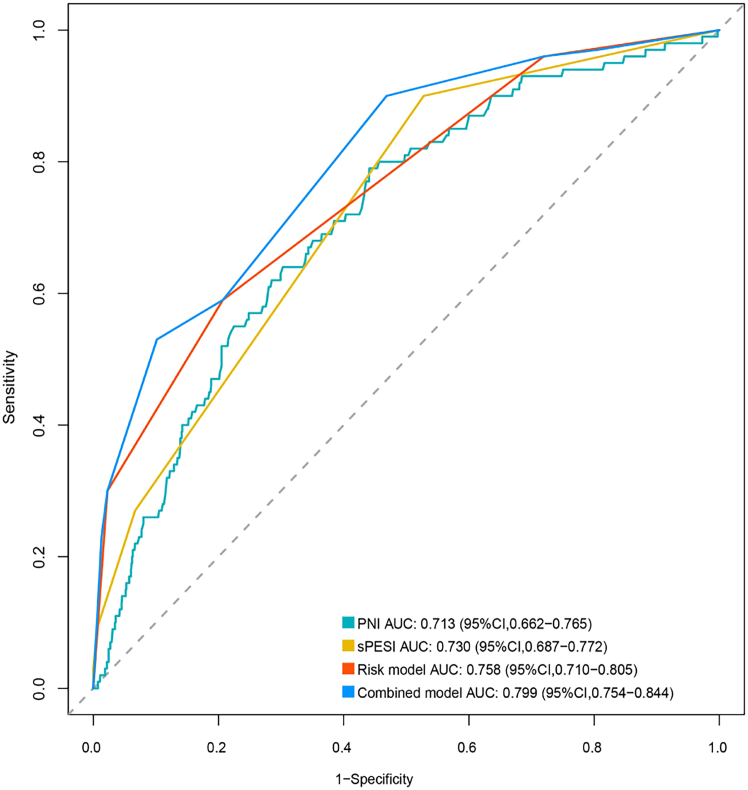
ROC curves of PNI, sPESI, ESC risk stratification, and combined model for 30-day mortality The AUC values with 95% confidence intervals are as follows: PNI, 0.713 (0.662–0.765); sPESI, 0.730 (0.687–0.772); ESC risk model, 0.758 (0.710–0.805); combined model, 0.799 (0.754–0.844). Statistical comparison by DeLong’s test confirmed that the combined model demonstrated a significant improvement over the ESC risk model (*p* < 0.001). The dashed line represents the reference (AUC = 0.5).

The performance of the PNI, sPESI, ESC risk stratification model, and combined model for predicting 30-day mortality in the discovery cohort was evaluated using ROC analysis (Figure 4). PNI alone demonstrated moderate discrimination, with an area under the curve (AUC) of 0.713 (95% CI 0.662–0.765) and a cutoff value of 42.54. At this threshold, the PNI had a sensitivity of 0.79 (95% CI 0.69–0.86), a specificity of 0.55 (95% CI 0.53–0.59), a positive predictive value (PPV) of 0.14 (95% CI 0.12–0.18), and a negative predictive value (NPV) of 0.97 (95% CI 0.95–0.98). sPESI alone had an AUC of 0.730 (95% CI 0.687–0.772); when a threshold of sPESI ≥1 was used, the sensitivity and specificity were 0.90 (95% CI 0.84–0.96) and 0.47 (95% CI 0.44–0.50), respectively. Its PPV and NPV were 0.14 (95% CI 0.11–0.16) and 0.98 (95% CI 0.97–0.99), respectively. The ESC risk stratification model alone achieved an AUC of 0.758 (95% CI: 0.710–0.805). When the intermediate-high risk threshold was used, the sensitivity and specificity were 0.79 (95% CI: 0.77–0.82) and 0.59 (95% CI: 0.49–0.69), respectively. The corresponding PPV and NPV were 0.21 (95% CI: 0.16–0.26) and 0.95 (95% CI: 0.94–0.97), respectively. For the combined model, patients were categorized as follows: low risk and PNI >42.54, low risk and PNI ≤42.54, intermediate-low risk and PNI >42.54, intermediate-low risk and PNI ≤42.54, intermediate-high risk and PNI >42.54, intermediate-high risk and PNI ≤42.54, high risk and PNI >42.54, and high risk and PNI ≤42.54. The combined model improved the predictive performance, achieving an AUC of 0.799 (95% CI 0.754–0.844). In this combined model, at the optimal cutoff value of intermediate-low risk and a PNI ≤42.54, the sensitivity and specificity were 0.90 (95% CI 0.84–0.96) and 0.53 (95% CI 0.50–0.56), respectively, with a PPV of 0.15 (95% CI 0.12–0.18) and an NPV of 0.98 (95% CI 0.97–0.99), respectively. The performance of the ESC risk stratification model combined with the PNI for predicting 30-day mortality in patients with APE was significantly better than that of the ESC risk stratification model alone (*p* < 0.001) (Figure 4; Table 4).

**Table 4:** Performance of the PNI, sPESI, ESE risk stratification model, and combined model for predicting 30-day mortality

Model	AUC (95% CI)	Cutoff	Sensitivity (95% CI)	Specificity (95% CI)	PPV (95% CI)	NPV (95% CI)
PNI	0.713 (0.662–0.765)	42.54	0.79 (0.69–0.86)	0.55 (0.53–0.59)	0.14 (0.12–0.18)	0.97 (0.95–0.98)
sPESI	0.730 (0.687–0.772)	1	0.90 (0.84–0.96)	0.47 (0.44–0.50)	0.14 (0.11–0.16)	0.98 (0.97–0.99)
ESC risk stratification model	0.758 (0.710–0.805)	Intermediate-high risk	0.79 (0.77–0.82)	0.59 (0.49–0.69)	0.21 (0.16–0.26)	0.95 (0.94–0.97)
Combined model	0.799 (0.754–0.844)	Intermediate-low risk and PNI ≤42.54	0.90 (0.84–0.96)	0.53 (0.50–0.56)	0.15 (0.12–0.18)	0.98 (0.97–0.99)

PNI, prognostic nutritional index; sPESI, simplified pulmonary embolism severity index; ESC, European Society of Cardiology.

In both internal and external validation cohorts, the combined model demonstrated a significantly higher AUC for predicting 30-day mortality than the ESC risk model alone (internal: 0.896 vs. 0.839; external: 0.792 vs. 0.746; both *p* < 0.05; Figures S2 and S3). This integration consistently enhanced sensitivity compared to the ESC risk model alone (internal: 0.93 [95% CI, 0.81–1.00] vs. 0.73 [0.51–0.96]; external: 0.96 [0.88–1.00] vs. 0.76 [0.59–0.93]), while maintaining high NPV (≥0.99), as shown in Table S4. These results indicate that the combination of PNI and the ESC risk model enhances both discrimination and accuracy when predicting 30-day mortality, compared with the ESC model alone.

### Enhanced risk stratification of intermediate-risk PE using the PNI

**Figure 5:**
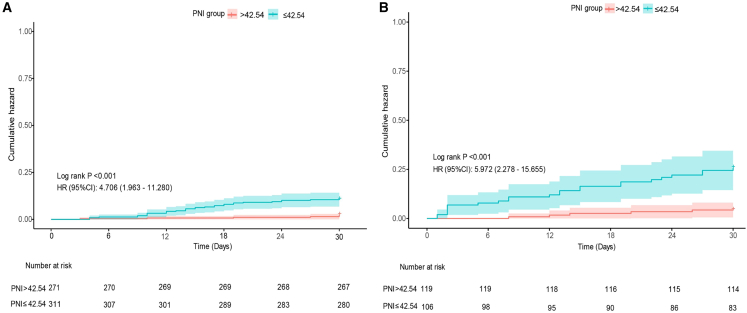
Kaplan-Meier curve for cumulative hazard stratified by PNI in the intermediate-risk group Patients were stratified into two groups: PNI >42.54 and PNI ≤42.54. (A) In the intermediate-low-risk group, the group with lower PNI had a significantly higher cumulative hazard (HR = 4.706, 95% CI: 1.963–11.280; log rank test, *p* < 0.001). (B) In the intermediate-high-risk group, a lower PNI was associated with an increased cumulative hazard (HR = 5.972, 95% CI: 2.278–15.655; log rank test, *p* < 0.001). The tables below detail the number of patients at risk over time for each group.

Among patients with intermediate-low-risk PE according to the ESC guidelines, those with a PNI ≤42.54 had a significantly higher cumulative risk of 30-day mortality than those with a PNI >42.54 did (HR 4.706, 95% CI 1.963–11.280; log rank *p* < 0.001). Similarly, in the intermediate-high-risk group, the risk of 30-day mortality was nearly 6-fold greater in patients with a PNI ≤42.54 than in patients with a PNI >42.54 (HR 5.972, 95% CI 2.278–15.655; log rank *p* < 0.001) (Figure 5). Among intermediate-low-risk patients, a PNI ≤42.54 predicted 30-day mortality, with a sensitivity of 0.84 (95% CI 0.72–0.96) and a specificity of 0.49 (95% CI 0.44–0.53). In intermediate-high-risk patients, a PNI ≤42.54 yielded a sensitivity of 0.83 (95% CI 0.69–0.97) and a specificity of 0.58 (95% CI 0.51–0.65) for predicting 30-day mortality. These findings indicate that the PNI provides additional prognostic stratification within the intermediate-risk of APE, allowing for more accurate prediction of short-term mortality.

## Discussion

In this study, we confirmed that the PNI is significantly inversely associated with both 30-day mortality and in-hospital mortality in patients with APE, suggesting its potential as a prognostic marker for short-term mortality. The PNI serves as an effective and reliable tool for identifying high-risk patients at admission, and its combination with ESC risk stratification model further improves the detection of those at greatest risk of death. Importantly, additional risk stratification using the PNI among intermediate-risk patients allowed a more accurate distinction of those at elevated risk of 30-day mortality. This may facilitate more effective triage, optimize the allocation of medical resources, and potentially guide decisions regarding the intensity of monitoring and interventions.

PNI, calculated from serum albumin levels and lymphocyte counts, serves as a useful marker that offers insights into a patient’s nutritional status, immune competence, and degree of chronic inflammation. Primarily applied in chronic diseases, PNI helps predict treatment response and clinical outcomes, including long-term mortality in specific conditions such as heart failure, atrial fibrillation, and cancer.^23^^,^^24^^,^^25^^,^^26^^,^^27^ Furthermore, it is associated with in-hospital mortality among COVID-19 patients who have cardiovascular risk factors.^28^ Previous studies have also highlighted the prognostic value of nutritional indices in patients with APE. For example, Hayiroglu et al.^22^ stratified 251 APE patients by PNI and reported significantly higher in-hospital mortality in the lowest PNI group, and PNI was an independent prognostic factor for survival of patients with APE. However, that study did not assess 30-day mortality. Consistent with and extending these findings, our study demonstrated that patients in the lowest PNI quartile (Q1) had significantly higher 30-day and in-hospital mortality than those in higher quartiles did. Moreover, the Q1 group was characterized by higher proportions of patients with elevated sPESI, cardiac troponin level, NT-proBNP level, and RVD, further supporting the link between low PNI and disease severity. Importantly, while the PNI correlated with severity, it also emerged as an independent predictor of both short-term outcomes, with its prognostic value being particularly pronounced in patients with coronary artery disease or type 2 diabetes, where significant interactions suggested amplified adverse effects of low PNI. Owing to increased protein catabolism and excretion coupled with reduced protein synthesis, patients with diabetes often have a negative nitrogen balance. This increases the risk of malnutrition, which in turn exacerbates insulin resistance, promotes systemic inflammation, and leads to adverse outcomes.^29^ In coronary heart disease, a low PNI is associated with poorer survival and reduced coronary flow.^30^ This occurs through two main pathways: hypoalbuminemia elevates cardiovascular risk by diminishing antioxidant and antithrombotic functions and increasing inflammation, while lymphopenia reflects impaired immunity that aggravates atherosclerotic disease.^31^^,^^32^

Notably, the predictive accuracy of the PNI for 30-day mortality was moderate (AUC 0.71), but its relatively high sensitivity indicates its potential utility for identifying high-risk patients, especially in the intermediate-risk subgroup, where clinical decision-making is most challenging. When the PNI was combined with the risk stratification model, the predictive value for 30-day mortality in patients with APE was greater than that for the risk stratification model alone (AUC: 0.799 vs. 0.758; *p* < 0.05), particularly by improving the sensitivity (0.90 vs. 0.79), thereby enabling more accurate identification of patients at high risk of death. Crucially, our findings address a critical gap in intermediate-risk APE stratification. A PNI ≤42.5 precisely identified a patient subgroup with markedly elevated mortality, corresponding to a 4.7-fold increase in 30-day mortality for intermediate-low-risk patients and a 6-fold increase for intermediate-high-risk patients compared to those with PNI >42.5.

Although the mechanisms by which these albumin-based composite markers of malnutrition influence clinical outcomes in patients with APE remain incompletely understood, several potential pathophysiological pathways have been proposed. A reduced PNI reflects an imbalance and insufficiency in the immunonutritional-inflammatory state. Since the PNI is derived from serum albumin levels and lymphocyte counts, its potential mechanisms can be interpreted from the perspectives of albumin and lymphocytes, respectively. Albumin is a multifunctional protein with key transporter, antioxidant, and anticoagulant activities and is a critical biomarker of systemic inflammation, nutritional status, and cardiovascular risk.^33^^,^^34^^,^^35^ Furthermore, albumin is essential for maintaining endothelial health and vascular tone by supporting nitric oxide bioavailability.^36^^,^^37^ However, as a negative acute-phase protein, its serum level decreases during inflammation, and hypoalbuminemia has been consistently associated with increased mortality.^38^ Furthermore, in APE, reduced cardiac output impairs hepatic perfusion and causes hypoxemia, resulting in ischemic-hypoxic liver injury and further suppression of albumin synthesis.^39^ This decline in albumin level diminishes its protective functions—including improving endothelial dysfunction, regulating vascular tone, inhibiting platelet aggregation, and maintaining endothelial permeability—thereby markedly increasing the risk of PE progression and adverse clinical outcomes.^36^^,^^40^ Research indicates that serum albumin levels exhibit a linear negative correlation with the risk of venous thromboembolism and can predict short-term outcomes in APE.^41^^,^^42^ Immune cells, particularly lymphocytes, are highly susceptible to nutritional deficiencies and decrease rapidly in the context of malnutrition. A reduced lymphocyte count, resulting in the limited functional activity of lymphocytes, impairs the initiation of an effective immune response, ultimately leading to unfavorable outcomes. Malnutrition and inflammation exhibit a bidirectional interplay.^43^ For example, hypoalbuminemia, a characteristic indicator of malnutrition, is strongly associated with systemic inflammation.^44^ Conversely, excessive inflammation suppresses albumin synthesis, perpetuating malnutrition and creating a self-reinforcing cycle that contributes to adverse outcomes.^45^

In light of these findings, PNI can be readily integrated into routine risk assessment for APE. We propose that hospital systems automatically compute and report PNI alongside traditional risk scores. This is particularly useful for stratifying intermediate-risk patients. A low PNI signals higher mortality and may justify closer monitoring or earlier therapy escalation, ultimately enhancing patient care and outcomes.

### Limitations of the study

Our study has several limitations. First, the analysis was limited to laboratory data at admission and did not capture dynamic changes that may offer more reliable information. Second, insufficient data on specific causes of death prevented distinction between mortality directly from PE and other causes. Finally, our external validation cohort was drawn from a single center and comprised a limited sample size. Therefore, in-depth subgroup analyses were not performed, as they would likely lack sufficient statistical power and could yield unstable findings. Future external validation in larger, multicenter prospective cohorts is warranted to further confirm the prognostic value of the PNI in patients with APE.

## Resource availability

### Lead contact

Further information and reasonable requests for resources should be directed to and will be addressed by the lead contact, Yimin Mao (yimin6107@haust.edu.cn).

### Materials availability

This study did not generate new unique reagents.

### Data and code availability

• The data cannot be made publicly accessible due to hospital regulations. Distributing these data without the necessary consent could potentially breach patient confidentiality and contravene the approval granted by the Institutional Review Board for this study. Any additional information required to reanalyze the data reported in this paper is available from the lead contact on request. Access to such data is available from the date of publication and requires a Data Access Agreement, which is examined and approved by the ethics committees who approved this research.
• This study did not generate an original code.
• Any additional information required for data reanalysis is available from the lead contact upon request.

## Acknowledgments

No funding was received for this work.

## Author contributions

S.L., S. Huang, W.W., P.G., and Y.M. contributed to the design of this study. S.L., S. Huang, W.W., B.C., C.M., S. Hou, and K.G. contributed to collecting and integrating the data. S.L., S. Huang, S. Hou, and J.Z. analyzed the results. S.L. and S. Huang wrote the manuscript. S. Hou, P.G., and Y.M. oversaw the study and contributed to critical revision of the manuscript. All authors reviewed and approved the final version.

## Declaration of interests

The authors declare no competing interests.

## STAR★Methods

### Key resources table

**Table:** 

REAGENT or RESOURCE	SOURCE	IDENTIFIER
**Software and algorithms**		

R 4.4.2	The R Foundation	https://www.r-project.org

**Other**		

Clinical and laboratory parameters	Electronic Medical Records	

### Experimental model and study participant details

#### Study design and population

This study included patients hospitalized for APE at two institutions. A retrospective cohort of patients admitted between January 2016 and June 2024 (*n*=1,654) from the First Affiliated Hospital of Henan University of Science and Technology served as the discovery cohort. An internal validation cohort consisted of a prospective cohort (n=213) enrolled at the same institution from July 2024 to June 2025. For external validation, a cohort of patients (n=267) hospitalized at Yanshi People’s Hospital between January 2022 and December 2024 was utilized. All the patients were Han Chinese from mainland China. This study was conducted in accordance with the Declaration of Helsinki and was approved by the ethics committees of the First Affiliated Hospital of Henan University of Science and Technology (Ethical Review No. 2024-150) and Yanshi People’s Hospital (Ethical Review No. 2025-0327). Written informed consent was obtained from all participants in the prospective cohorts. For the retrospective cohort, the requirement for informed consent was waived by the respective Ethics Committees.

Eligible patients were adults aged over 18 years with objectively confirmed APE who were diagnosed by computed tomography pulmonary angiography (CTPA), ventilation-perfusion (V/Q) scans, or pulmonary angiography. Right ventricular function was assessed using transthoracic echocardiography. Patients were excluded if they met any of the following criteria: (1) unconfirmed PE; (2) inability to perform ESC risk stratification; (3) missing parameters required for PNI calculation within 24 hours of hospital admission; or (4) age under 18 years.

Patients were diagnosed with acute RVD if they met at least one of the following criteria: (1) right ventricular enlargement, defined as an end-diastolic diameter >30 mm in the apical four-chamber view or a right-to-left ventricular end-diastolic diameter ratio >0.9; (2) paradoxical septal motion; or (3) a peak tricuspid regurgitant jet velocity exceeding 2.8 m/s.^46^ According to the ESC risk stratification model,^7^ patients were classified as low risk if they had an sPESI score of 0 with no evidence of RVD and no elevation in cardiac troponin levels. Patients were considered intermediate-low risk if they had an sPESI score of 0 with either RVD or positive cardiac troponin levels (or both) or if they had an sPESI score ≥1 without RVD or cardiac troponin elevation. Patients were classified as intermediate-high risk if they had an sPESI score ≥1 in combination with both RVD and elevated cardiac troponin levels. Haemodynamic instability, in combination with PE confirmation on CTPA and evidence of RVD on echocardiography, is sufficient to classify a patient as having high-risk PE. The PNI was calculated using the following formula: serum albumin concentration (g/L) + 5 × total lymphocyte count (10^9^/L).

### Method details

#### Data collection

Data were collected from the hospital information system for patients who met the study’s inclusion and exclusion criteria. We collected data on demographic variables (age, sex, weight), vital signs (heart rate, systolic blood pressure, respiratory rate, partial pressure of oxygen [PaO_2_], partial pressure of carbon dioxide [PaCO_2_]), comorbidities (sepsis, cancer, hypertension, etc.), laboratory parameters (white blood cell [WBC] count, haemoglobin [HGB] level, platelet [PLT] count, N-terminal pro-brain natriuretic peptide [NT-proBNP], cardiac troponin [cTn], etc.), echocardiographic assessments (right ventricular dysfunction), sPESI (Table S1), 2019 ESC risk stratification, therapeutic interventions (thrombolysis, catheter-based therapy), and clinical outcomes, including 30-day mortality and in-hospital mortality.

#### Follow-up and outcome

In our study, the primary outcome was 30-day all-cause mortality, defined as death from any cause within 30 days of admission. The secondary outcome was in-hospital mortality, defined as death from any cause during hospitalization. Follow-up was conducted using a combination of hospital admission records, telephone interviews, and outpatient consultations.

### Quantification and statistical analysis

#### Statistical analysis

Variables with less than 20% missing data were imputed via multiple imputation by chained equations (MICE) to minimize potential bias. For normally distributed data, continuous variables are expressed as the means±standard deviations, whereas nonnormally distributed data are presented as medians with interquartile ranges. Differences in normally distributed variables were assessed using Student’s t test or analysis of variance (ANOVA). For nonnormally distributed variables, the Mann‒Whitney U test or Kruskal‒Wallis test was applied. Categorical variables are reported as frequencies and percentages and were compared using the chi-square test or Fisher’s exact test.

The incidence of endpoint events across different PNI levels was evaluated using Kaplan‒Meier survival analysis, and differences were compared with the log-rank test. Cox proportional hazards regression was employed to calculate hazard ratios (HRs) and 95% confidence intervals (CIs) for the association between the PNI and 30-day mortality. Logistic regression was used to estimate odds ratios (ORs) and 95% CIs for in-hospital mortality. PNI was analysed both as a continuous variable and as quartiles. Adjusted multivariate models were developed to assess the prognostic impact of the PNI. Three models were constructed: Model 1, univariate analysis; Model 2, adjusted for age, sex, and body weight; and Model 3, adjusted for Model 2 covariates plus heart rate, respiratory rate, systolic blood pressure, PaO_2_, PaCO_2_, haemoglobin, platelet count, neutrophil count, D-dimer, alanine aminotransferase (ALT), aspartate aminotransferase (AST), lactate, creatinine, blood urea nitrogen (BUN), sPESI score, positive cardiac troponin, positive NT-proBNP, RVD, sepsis, heart failure, chronic pulmonary disease (CPD), coronary artery disease (CAD), hypertension, type 2 diabetes, chronic kidney disease (CKD), and cancer. The variance inflation factor (VIF) was used to evaluate multicollinearity; when the VIF is less than 5, it is typically interpreted as the absence of substantial multicollinearity among the variables.

To explore potential nonlinear relationships between the PNI and mortality, restricted cubic spline (RCS) regression was performed. Subgroup analyses were conducted to assess heterogeneity across different strata, including age (<65 vs. ≥65 years); sex; the presence of sepsis, cancer, hypertension, type 2 diabetes, CAD, heart failure, CKD, and CPD; and APE risk stratification.

Receiver operating characteristic (ROC) curves were constructed to assess the predictive value of the sPESI score, PNI, ESC risk stratification model, and ESC risk stratification model combined with the PNI for 30-day mortality in APE patients. The areas under the ROC curves (AUCs) were compared using DeLong’s test. Finally, we further investigated the prognostic utility of the PNI in patients with intermediate-low and intermediate-high risk APE, as defined by the ESC risk stratification model.

All the statistical analyses were performed using R software (version 4.2.2; https://www.r-project.org). A two-sided *P* value < 0.05 indicated statistical significance.
