# Supplementary material for: The prognostic nutritional index improves risk stratification for acute pulmonary embolism
Source: iScience. 2026 Jan 2;29(2):114623. doi: 10.1016/j.isci.2025.114623 (PMC12834838; doi:10.1016/j.isci.2025.114623)
Supplement: Document S1. Figures S1–S3 and Tables S1–S4 [file mmc1.pdf]

iScience, Volume *29*

Supplemental information

The prognostic nutritional index improves risk stratification for acute pulmonary embolism

Shuangping Li, Shenshen Huang, Wei Wang, Jing Zhang, Bo Chen, Kelei Guo, Chenglong Ma, Shuaihui Hou, Pengfei Gao, and Yimin Mao

Table S1. sPESI score

Variables	Score
Age > 80 years	1
Systolic blood pressure (SBP) < 100 mmHg	1
Pulse oxygen saturation (SpO2)< 90%,	1
Heart rate ≥ 110 bpm	1
History of cancer	1
History of chronic cardiopulmonary disease	1

Table S2. Baseline characteristics and comparisons between 30-day survivors and non-survivors in internal validation cohort

Variables	Total (n = 208)	30-day survival (n = 193)	30-day mortality (n = 15)	*P*
**Demographic variables**				
Age, years	71.00 (63.00, 76.00)	71.00 (62.00, 76.00)	74.00 (68.50, 87.00)	0.021
Weight, Kg	73.00 (63.00, 85.00)	73.00 (63.00,85.00)	85.00 (65.00, 105.00)	0.079
Male, n(%)	102 (49.04)	91 (47.15)	11 (73.33)	0.051
**Vital signs**				
Heart rate, bpm	85.00 (76.00, 96.25)	85.00 (76.00, 96.00)	88.00 (77.50, 105.50)	0.508
SBP, mmHg	131.00 (115.75, 144.00)	132.00 (116.00,144.00)	100.00 (86.50,141.00)	0.009
RR, bpm	20.00 (19.00, 21.00)	20.00 (19.00, 21.00)	20.00 (18.00, 22.50)	0.789
PO_2, mmHg	76.40 (65.00, 95.70)	77.00 (65.40, 96.00)	76.30 (62.00, 80.40)	0.305
PCO_2, mmHg	35.20 (31.58, 39.58)	35.40 (31.70,39.80)	32.00 (27.25, 39.00)	0.082
**Comorbidities**				
Sepsis, n(%)	10 (4.81)	6 (3.11)	4 (26.67)	0.003
Cancer, n(%)	44 (21.15)	38 (19.69)	6 (40.00)	0.127
Hypertension, n(%)	61 (29.33)	59 (30.57)	2 (13.33)	0.264
CAD, n(%)	33 (15.87)	31 (16.06)	2 (13.33)	1.00
Heart failure, n(%)	6 (2.88)	5 (2.59)	1 (6.67)	0.365
CKD, n(%)	6 (2.88)	5 (2.59)	1 (6.67)	0.365
Diabetes, n(%)	29 (13.94)	27 (13.99)	2 (13.33)	1.000
CPD, n(%)	13 (6.25)	12 (6.22)	1 (6.67)	1.000
**Laboratory tests**				
WBC, $\times10^9$/L	7.98 (6.25, 10.23)	7.81 (6.09, 9.93)	10.19 (8.29, 13.81)	0.029
HGB, g/L	128.00 (112.75, 142.00)	129.00 (113.00, 142.00)	108.00 (105.50, 142.50)	0.216
Platelet, $\times10^9$/L	193.5 (154.00, 250.50)	194.00 (157.00, 250.00)	171.00 (69.50, 249.50)	0.151
Neutrophil, $\times10^9$/L	6.09 (4.24, 8.07)	6.00 (4.12, 7.67)	8.57 (6.68, 12.32)	0.009
Lymphocyte, $\times10^9$/L	1.25 (0.89, 1.62)	0.86 (0.59, 1.15)	0.86 (0.59, 1.15)	0.006
Albumin, g/L	38.70 (35.20, 41.90)	38.90 (36.10, 42.00)	33.10 (28.65, 34.90)	<.001
PNI	45.58 (40.24, 49.14)	45.80 (41.40, 50.20)	35.75 (33.15, 39.28)	<.001
Lactate, mmol/L	1.47 (1.10, 1.97)	1.43 (1.10, 1.90)	1.68 (1.40, 2.00)	0.234
D-Dimer, mg/L	3.52 (1.28, 8.54)	3.40 (1.26, 8.44)	3.86 (2.25, 12.48)	0.346
Total bilirubin, μmol/L	10.70 (8.30, 14.93)	10.60 (8.20, 14.30)	17.60 (9.10, 19.85)	0.047
Direct bilirubin, μmol/L	3.40 (2.30, 5.03)	3.20 (2.30, 4.70)	7.80 (5.15, 9.55)	<.001
ALT, U/L	21.00 (14.00, 34.00)	20.00 (14.00, 31.00)	27.00 (20.50, 41.00)	0.139
AST, U/L	21.00 (17.00, 30.00)	21.00 (17.00, 29.00)	30.00 (27.00, 37.50)	0.021
Glucose, mmol/L	6.18 (5.12, 7.99)	6.12 (5.13, 7.94)	6.26 (5.07, 8.66)	0.838
BUN, mmol/L	6.00 (4.60, 8.30)	5.90 (4.60, 8.20)	7.60 (5.80, 15.95)	0.013
Creatinine, μmol/L	66.00 (52.00, 84.75)	64.00 (52.00, 83.00)	70.00 (59.00, 155.00)	0.063
**Severity**				

Variables	Total (n = 208)	30-day survival (n = 193)	30-day mortality (n = 15)	*P*
sPESI	1.00 (0.00, 1.00)	1.00 (0.00, 1.00)	2.00 (0.00, 2.00)	<.001
Positive cardiac troponin，n (%)	52 (25.00)	40 (20.73)	12 (80.00)	<.001
Positive NT-proBNP , n (%)	108 (51.92)	94 (48.70)	14 (93.33)	<.001
RVD, n(%)	86 (41.35)	75 (38.86)	11 (73.33)	0.009
Risk stratification, n(%)				<.001
Low risk	56 (26.92)	55 (28.50)	1 (6.67)	
Intermediate-low risk	116 (55.77)	113 (58.55)	3 (20.00)	
Intermediate-high risk	25 (12.02)	22 (11.40)	3 (20.00)	
High risk	11 (5.29)	3 (1.55)	8 (53.33)	
**Therapy**				
rt-PA, n(%)	4 (1.92)	4 (2.07)	0 (0.00)	1.000
Catheter-based therapy, n(%)	13 (6.25)	13 (6.74)	0 (0.00)	0.605

SBP, systolic blood pressure; RR, respiratory rate; PCO_2, partial pressure of carbon dioxide; PO_2, partial pressure of oxygen; WBC, white blood cell; HGB, haemoglobin; BUN, blood urea nitrogen; ALT, alanine aminotransferase; AST, aspartate aminotransferase; PNI, prognostic nutritional index; sPESI, simplified pulmonary embolism severity index; CAD, coronary artery disease; CPD, chronic pulmonary disease; CKD, chronic kidney disease; NT-proBNP, N-terminal pro–B-type natriuretic peptide; RVD, right ventricular dysfunction; rt-PA, recombinant tissue-type plasminogen activator

Table S3. Baseline characteristics and comparisons between 30-day survivors and non-survivors in external validation cohort

Variables	Total (n = 212)	30-day survival (n = 187)	30-day mortality (n = 25)	*P*
**Demographic variables**				
Age, years	63.00 (49.00, 71.00)	63.00 (48.00, 70.00)	68.00 (56.50, 74.00)	0.050
Weight, Kg	61.00 (53.00, 75.00)	61.00 (53.00, 75.00)	61.00 (55.00, 70.00)	0.761
Male, n(%)	110 (51.89)	96 (51.34)	11 (56.00)	0.661
**Vital signs**				
Heart rate, bpm	84.00 (74.00, 98.00)	83.00 (73.00, 96.00)	104.00 (82.00, 110.00)	0.002
SBP, mmHg	126.50 (110, 138.00)	127.00 (112.00,139.00)	125.00 (87.00, 135.00)	0.055
RR, bpm	20.00 (20.00, 21.25)	20.00 (20.00, 21.00)	20.00 (18.00, 24.00)	0.735
PO_2, mmHg	85.80 (68.65, 109.25)	87.00 (72.95, 113.00)	59.00 (50.00, 81.00)	<.001
PCO_2, mmHg	36.50 (32.00, 40.52)	36.00 (31.90, 40.00)	45.00 (36.00, 56.00)	<.001
**Comorbidities**				
Sepsis, n(%)	19 (8.96)	15 (8.02)	4 (16.00)	0.348
Cancer, n(%)	27 (12.74)	24 (12.83)	3 (12.00)	1.000
Hypertension, n(%)	62 (29.25)	55 (29.41)	7 (28.00)	0.884
CAD, n(%)	19 (8.96)	17 (9.09)	2 (8.00)	1.000
Heart failure, n(%)	3 (1.42)	2 (1.07)	1 (4.00)	0.315
CKD, n(%)	13 (6.13)	9 (4.81)	4 (16.00)	0.081
Diabetes, n(%)	25 (11.79)	24 (12.83)	1 (4.00)	0.339
CPD, n(%)	14 (6.60)	11 (5.88)	3 (12.00)	0.467
**Laboratory tests**				
WBC, $\times 10^9$/L	8.27 (6.12, 10.75)	7.70 (6.02, 10.12)	12.00 (10.49, 16.43)	<.001
HGB, g/L	129.00 (115.00, 140.00)	128.00 (118.00, 140.00)	129.00 (103.00, 136.00)	0.286
Platelet, $\times 10^9$/L	206.5 (165.75, 261.00)	206.00 (165.50, 259.50)	218.00 (175.00, 301.00)	0.294
Neutrophil, $\times 10^9$/L	5.50 (3.73, 8.23)	5.13 (3.65, 7.55)	10.07 (8.00, 13.62)	<.001
Lymphocyte, $\times 10^9$/L	1.50 (1.14, 1.98)	1.52 (1.19, 2.00)	1.16 (0.63, 1.59)	0.005
Albumin, g/L	35.61 (32.80, 39.41)	36.12 (33.40, 39.50)	32.50 (28.10, 36.70)	0.006
PNI	43.48 (39.84, 47.85)	43.90 (40.30, 48.60)	38.25 (33.35, 42.99)	<.001
Lactate, mmol/L	1.3 (1.00, 1.90)	1.20 (0.90, 1.70)	4.30 (1.50, 6.60)	<.001
D-Dimer, mg/L	3.83 (1.88, 10.40)	3.64 (1.93, 9.81)	7.94 (1.54, 12.98)	0.322
Total bilirubin, μmol/L	12.00 (9.10, 16.47)	11.40 (9.00, 15.40)	18.90 (12.00, 22.40)	<.001
Direct bilirubin, μmol/L	2.70 (1.67, 4.40)	2.50 (1.60, 4.10)	6.20 (2.80, 8.70)	<.001
ALT, U/L	24.00 (15.75, 41.25)	23.00 (14.00, 39.50)	40.00 (28.00, 64.00)	0.002
AST, U/L	25.00 (20.00, 39.00)	24.00 (19.00, 36.50)	34.00 (24.00, 57.00)	0.005
Glucose, mmol/L	6.04 (5.07, 7.75)	5.94 (5.00, 7.55)	7.00 (5.50, 9.15)	0.044
BUN, mmol/L	4.98 (3.79, 6.81)	4.73 (3.70, 6.43)	6.58 (5.16, 11.80)	<.001
Creatinine, μmol/L	77.00 (62.00, 93.00)	75.90 (62.09, 91.05)	86.52 (60.52, 130.00)	0.109
**Severity**				

Variables	Total (n = 212)	30-day survival (n = 187)	30-day mortality (n = 25)	*P*
sPESI	0.00 (0.00, 1.00)	0.00 (0.00, 1.00)	1.00 (1.00, 1.00)	<.001
Positive cardiac troponin，n (%)	112 (52.8)	93 (48.73)	19 (76.00)	0.010
Positive NT-proBNP , n (%)	139 (65.57)	115 (61.50)	24 (96.00)	<.001
RVD, n(%)	120 (56.60)	98 (52.41)	22 (88.00)	<.001
Risk stratification, n(%)				<.001
Low risk	33 (15.57)	32 (17.11)	1 (4.00)	
Intermediate-low risk	99 (46.70)	94 (50.27)	5 (20.00)	
Intermediate-high risk	70 (33.02)	56 (29.95)	14 (56.00)	
High risk	10 (4.72)	5 (2.67)	5 (20.00)	
**Therapy**				
Reperfusion therapy, n(%)	34 (16.04)	21 (11.23)	13 (52.00)	<.001

SBP, systolic blood pressure; RR, respiratory rate; PCO_2, partial pressure of carbon dioxide; PO_2, partial pressure of oxygen; WBC, white blood cell; HGB, haemoglobin; BUN, blood urea nitrogen; ALT, alanine aminotransferase; AST, aspartate aminotransferase; PNI, prognostic nutritional index; sPESI, simplified pulmonary embolism severity index; CAD, coronary artery disease; CPD, chronic pulmonary disease; CKD, chronic kidney disease; NT-proBNP, N-terminal pro–B-type natriuretic peptide; RVD, right ventricular dysfunction

Table S4. Comparison of predictive performance between the ESC risk model and the combined model for 30-day mortality

Cohort	Model	AUC (95%CI)	Sensitivity (95% CI)	Specificity (95% CI)	PPV (95% CI)	NPV (95% CI)
Internal	ESC risk	0.840 (0.710-0.969)	0.73 (0.51–0.96)	0.87 (0.82–0.92)	0.31 (0.15–0.45)	0.98 (0.95–0.99)
	Combined	0.896 (0.795-0.988)	0.93 (0.81–1.00)	0.66 (0.59–0.73)	0.18 (0.09–0.26)	0.99 (0.98–1.00)
External	ESC risk	0.746 (0.649-0.844)	0.76 (0.59–0.93)	0.67 (0.61–0.74)	0.24 (0.14–0.33)	0.95 (0.92–0.99)
	Combined	0.792 (0.705-0.878)	0.96 (0.88–1.00)	0.48 (0.40–0.55)	0.20 (0.13–0.27)	0.99 (0.97–1.00)

Esc, European Society of Cardiology

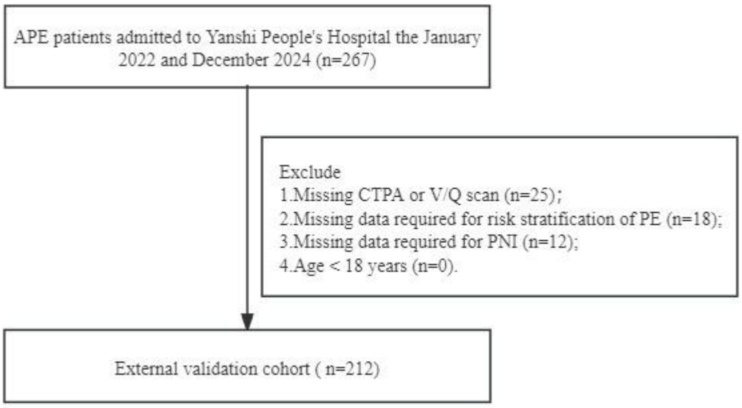

Figure S1. Flowchart of patient selection in external validation cohort.

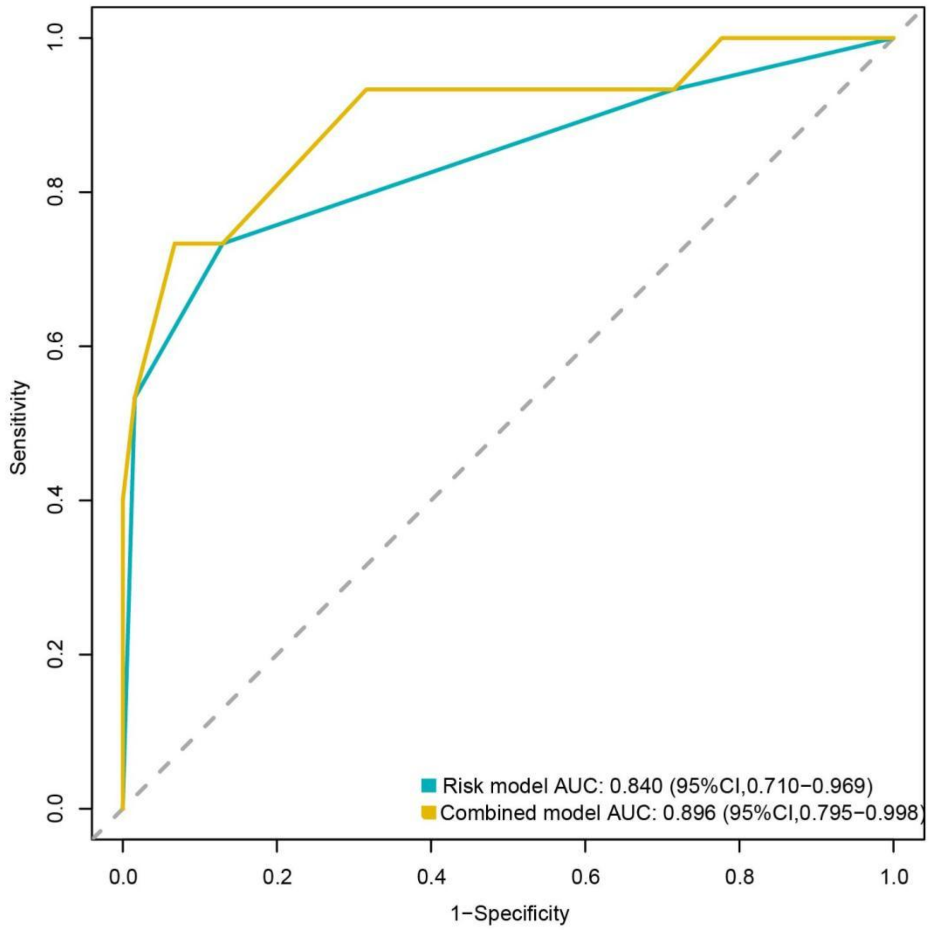

Figure S2. ROC curves of ESC risk stratification and combined model for 30-day mortality in internal validation cohort. The AUC for the ESC risk model was 0.840 (95% [CI]: 0.710–0.969). The combined model achieved an AUC of 0.896 (95% CI: 0.795–0.998). The difference in AUC between the two models was statistically significant by DeLong's test (*P* < 0.05).

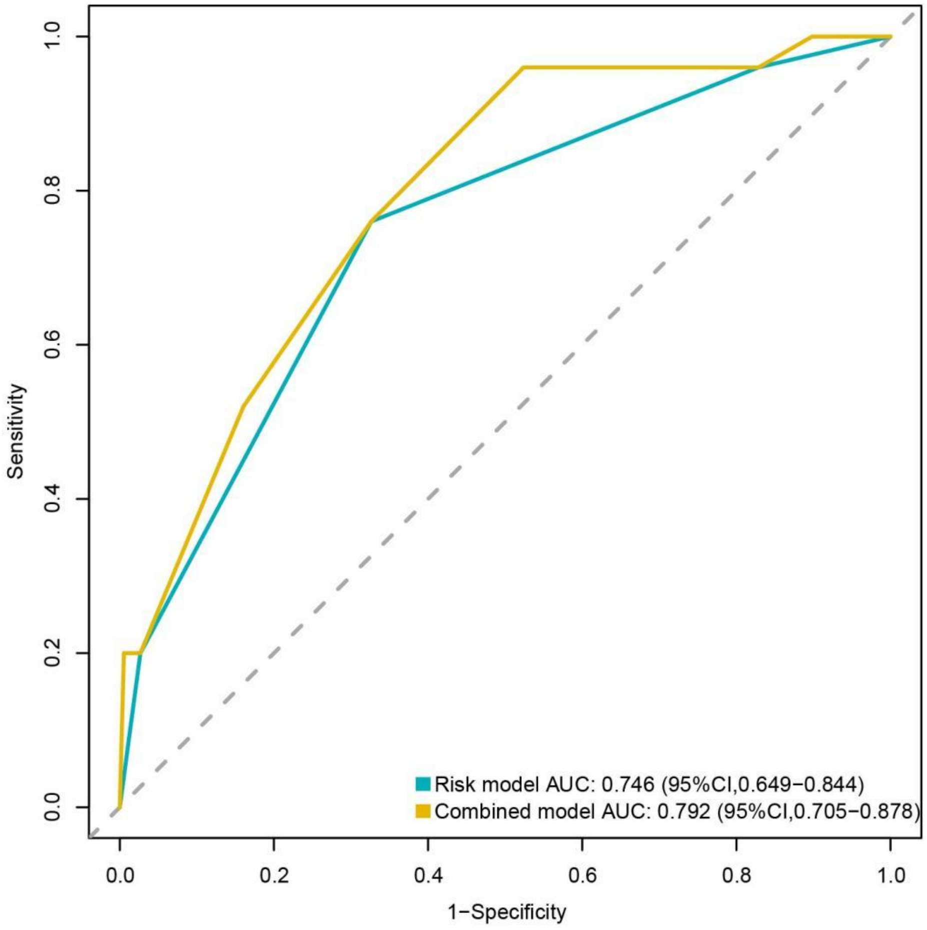

Figure S3. ROC curves of ESC risk stratification and combined model for 30-day mortality in external validation cohort. The AUC for the ESC risk model was 0.746 (95% [CI]: 0.649–0.844). The combined model achieved an AUC of 0.792 (95% CI: 0.705–0.878). The difference in AUC between the two models was statistically significant by DeLong's test ($P < 0.05$).
